# Community-acquired carbapenem-resistant *Acinetobacter baumannii* pneumonia in an elderly woman with chronic kidney disease: A case report

**DOI:** 10.1016/j.idcr.2026.e02584

**Published:** 2026-04-22

**Authors:** Hasaan Salman Shafi, Omair Farooq

**Affiliations:** a Al-Aleem Medical College, F8JV+R2J, Ferozepur Road, Opposite Arfa Kareem Tower, Nishtar Town, Lahore, Pakistan; b Akhtar Saeed Medical & Dental College, 95CR+V7V, 80' Blvd, Tulip Extension Tulip Block Sector C Bahria Town, Lahore, Pakistan

**Keywords:** *Acinetobacter baumannii*, Community-acquired pneumonia, Multidrug resistance, Carbapenem resistance, Bronchoalveolar lavage, Aminoglycosides, Case report

## Abstract

Carbapenem-resistant *Acinetobacter baumannii* (CRAB) is a major cause of hospital-acquired infections but is rarely implicated in community-acquired pneumonia (CAP). We report the case of a 79-year-old woman with stage 3 chronic kidney disease who presented with severe CAP and respiratory failure. Bronchoscopy with bronchoalveolar lavage (BAL) identified CRAB sensitive to tobramycin and levofloxacin but resistant to all Beta-lactams and carbapenems. She was successfully treated with intravenous tobramycin and levofloxacin, followed by oral doxycycline and levofloxacin. Clinical and radiologic resolution was achieved by week 6. This case highlights a rare instance of community-acquired CRAB pneumonia and underscores the importance of BAL-based diagnosis and susceptibility-guided therapy, particularly in high-risk patients.

## Introduction

Carbapenem-resistant *Acinetobacter baumannii* (CRAB) has emerged as a formidable cause of nosocomial pneumonia, particularly in elderly and critically ill patients [1]. Infections due to CRAB are exceedingly difficult to treat and carry alarmingly high mortality rates, with recent studies reporting case fatality often exceeding 50% [2], [3]. This pathogen exhibits extensive resistance to multiple antibiotic classes, leaving clinicians with very few effective therapeutic options [1]. In some regions, virtually all A. baumannii isolates are resistant to carbapenems and frequently co-resistant to other last-line agents (e.g., colistin and sulbactam. Consequently, the management of severe CRAB pneumonia often relies on various synergistic combination regimens, despite a lack of robust clinical evidence to guide specific drug choices [1].

In this report, we describe a rare case of community-acquired CRAB pneumonia in an elderly woman with stage 3 chronic kidney disease.

## Case presentation

A 79-year-old woman with a known history of stage 3 chronic kidney disease (CKD) presented to the emergency department with complaints of cough and progressive dyspnea. On admission, she was hemodynamically stable (HR: 94 bpm, BP: 126/63 mmHg) and afebrile with a weight of 70 kg. Auscultation revealed bronchial breath sounds on the right side. There was no peripheral edema. Laboratory tests showed marked systemic inflammation: C-reactive protein (CRP) > 200 mg/L, erythrocyte sedimentation rate (ESR) 85 mm/h, total leukocyte count (TLC) 31,000/µL, urea 85 mg/dL, and serum creatinine 2.1 mg/dL. Blood and urine cultures, as well as AFB smear, were negative.

**Fig. 1:**
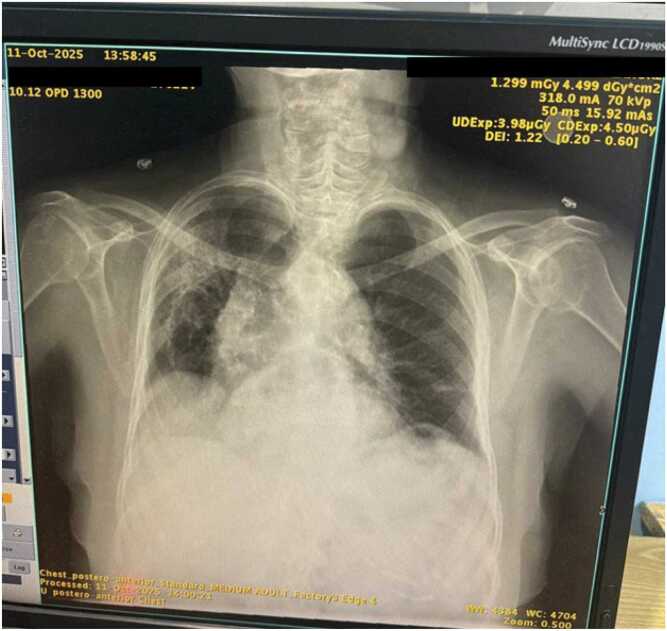
Chest X-ray on admission (11 October 2025): shows bilateral patchy consolidations predominantly in lower zones, consistent with severe pneumonia.

**Fig. 2:**
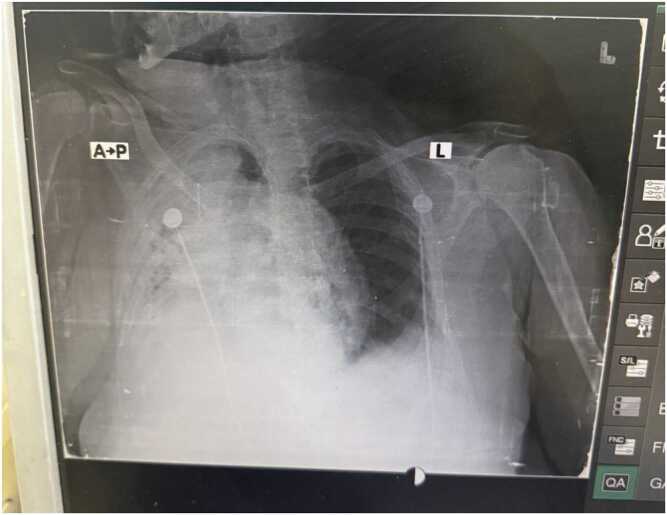
Chest X-ray during hospitalization (approx. 2nd September): persistent diffuse opacities, worsening consolidation.

A clinical diagnosis of severe community-acquired pneumonia (CAP) was made. She was started empirically on intravenous cefoperazone-sulbactam (2 g BID), later escalated to meropenem (1 g BID), moxifloxacin (400 mg daily), linezolid (600 mg BID), intravenous methylprednisolone (40 mg BID), and supportive intravenous fluids. Initial imaging revealed diffuse bilateral consolidation (Fig. 1), with progression seen on follow-up imaging (Fig. 2)

Due to worsening respiratory status and persistent hypoxemia requiring BiPAP support with 15 L/min oxygen, a bronchoscopy was performed on day 2 of hospitalization. Findings included diffuse mucopurulent secretions and significant inflammation in all lobes. The right middle lobe was severely inflamed with only 15–20% lumen patency. Bronchoalveolar lavage (BAL) was performed, and cultures were sent for bacterial, fungal, and TB testing.

By day 4, leukocyte count had decreased to 18,000/µL, and creatinine improved to 1.5 mg/dL. On day 5, BAL cultures returned positive for heavy growth of Acinetobacter baumannii, which was resistant to all beta-lactams and carbapenems but sensitive to tobramycin, levofloxacin, and minocycline, and intermediately sensitive to doxycycline (Table 1). All other antibiotics were stopped, and she started on intravenous tobramycin (200 mg IV stat then 140 mg twice a day) and levofloxacin. Chest physiotherapy was initiated daily.

**Table 1:** Antibiotic sensitivity profile.

**Antibiotic Class**	**Antibiotic**	**Susceptibility**
Anti-folates	Co-trimoxazole	Resistant
Fluoroquinolones	Ciprofloxacin	Resistant
Fluoroquinolones	Levofloxacin	Sensitive
Phenicols	Chloramphenicol	Resistant
Cephalosporins	Cefepime	Resistant
Cephalosporins	Ceftriaxone	Resistant
Cephalosporins	Cefotaxime	Resistant
Cephalosporins	Cefoperazone/Sulbactam	Resistant
Aminoglycosides	Tobramycin	Sensitive
Aminoglycosides	Gentamicin	Resistant
Aminoglycosides	Amikacin	Resistant
Tetracyclines	Minocycline	Sensitive
Tetracyclines	Doxycycline	Intermediate
Carbapenems	Meropenem	Resistant
Carbapenems	Imipenem	Resistant
Aminopenicillins	Piperacillin/Tazobactam	Resistant

S = Sensitive, I = Intermediate, R = Resistant.

By day 9, leukocyte count improved further to 11,000/µL, and creatinine rose slightly to 1.6 mg/dL. She was discharged on day 13, at which point creatinine was 1.9 mg/dL while TLC was 12k/µL with ongoing therapy: 5 days of IV tobramycin and levofloxacin. The patient was switched to Oral doxycycline (100 mg BID) and levofloxacin (500 mg daily) on subsequent OPD follow-up.

At outpatient follow-up on day 16, inflammatory markers remained elevated (TLC 11.9k/µL, CRP 142 mg/L), with a creatinine of 2.2 mg/dL. By day 21, inflammatory markers were improving (TLC 8.3k/µL, CRP 103 mg/L), though creatinine peaked at 2.6 mg/dL. Tobramycin was discontinued, and oral doxycycline and levofloxacin were continued.

**Fig. 3:**
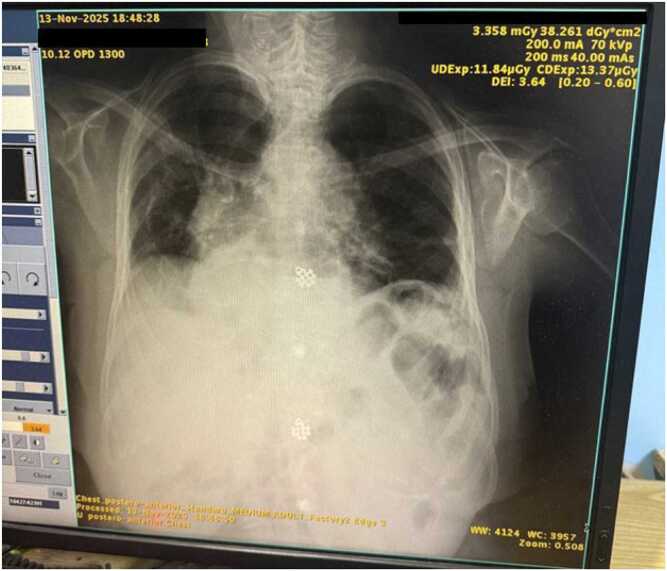
Follow-up chest X-ray (13 November 2025): marked radiological improvement with resolution of consolidations.

**Fig. 4:**
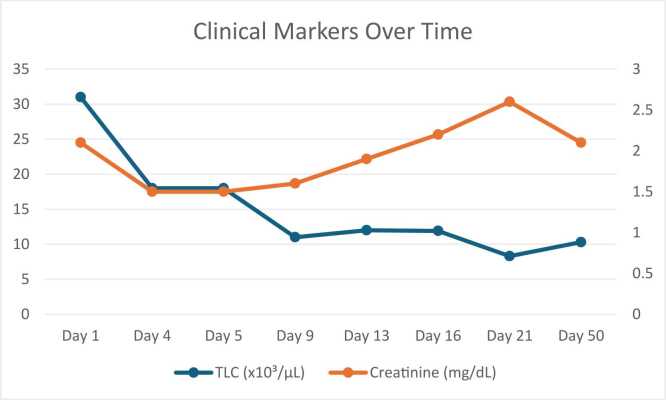
Clinical Markers Over Time.

**Fig. 5:**
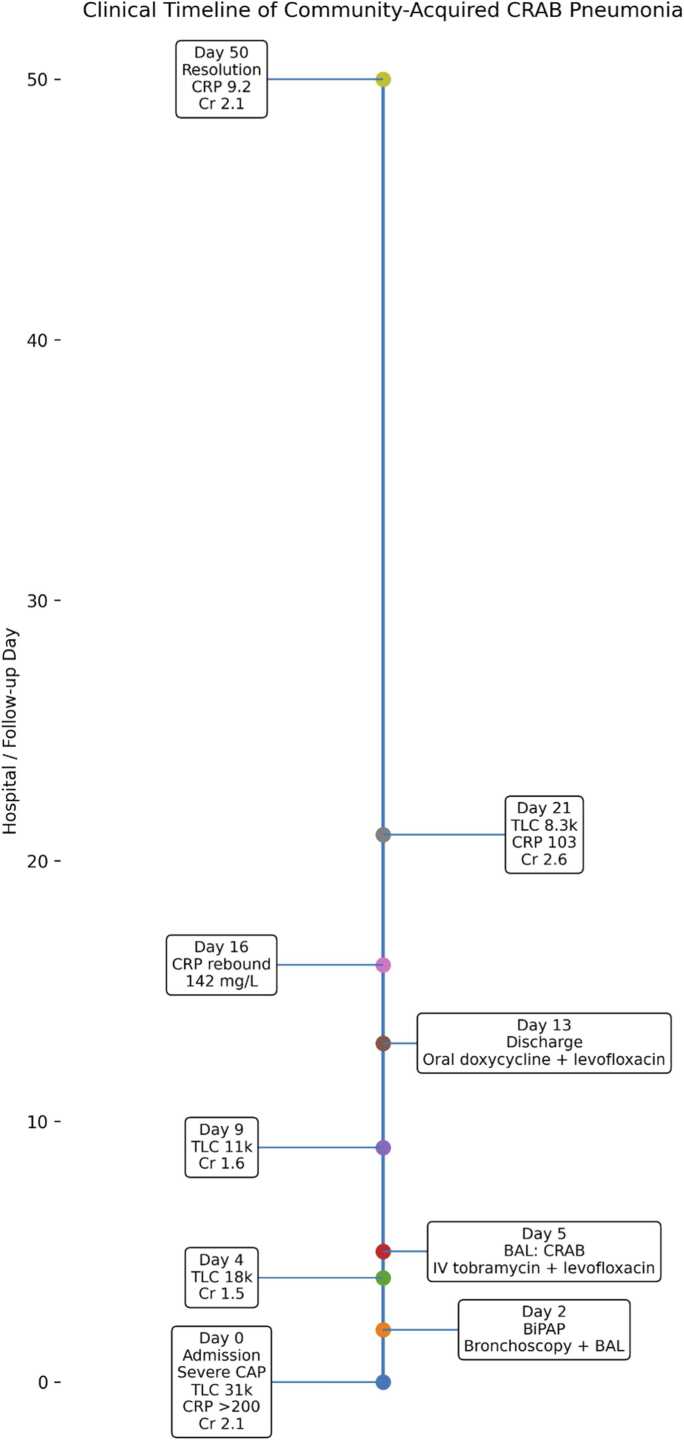
Clinical Timeline.

Over the following weeks, the patient continued to improve clinically. By day 50, TLC normalized to 10.3k/µL, CRP dropped to 9.2 mg/L, and creatinine stabilized at 2.1 mg/dL. A total of 6 weeks of doxycycline and levofloxacin therapy was completed. Follow-up chest imaging demonstrated marked resolution of prior bilateral consolidations (Fig. 3). The patient remained asymptomatic at the end of therapy. Fig. 4 shows clinical markers over time. Fig. 5 shows a visual timeline.

## Discussion

Carbapenem-resistant *Acinetobacter baumannii* (CRAB) has emerged as a significant healthcare-associated infection worldwide and is associated with high mortality rates and difficult-to-treat infections. [4]. The WHO has classified CRAB as a critical priority pathogen due to its multidrug resistance [4]. In hospital settings, CRAB is associated with severe infections and mortality rates reaching 50–70% in some cohorts [4]. emphasizing its major clinical and epidemiologic significance.

In contrast, community-acquired A. baumannii pneumonia is exceedingly rare. Globally, A. baumannii accounts for less than 10% of bacterial pneumonia cases, even in subtropical regions where it has been reported, and cases arising in temperate climates or outside hospital settings are extremely uncommon [5]. When community acquired infection does occur, it tends to be severe with a rapid progression. The resistant nature of the pathogen results in delayed effective therapy, further worsening outcomes. These infections typically affect patients with previous comorbidities such as diabetes, chronic kidney disease, or chronic lung disease; however, previously healthy individuals can also be affected [5]. Overall, the rarity and severity of community-acquired CRAB pneumonia make each case clinically notable.

Compared to prior reports, the present case is unusual in its outcome and management. Most documented cases of community-acquired A. baumannii pneumonia have had an aggressive clinical course with early septic shock, acute respiratory failure, and poor outcomes. In a large Asian series, 92% of patients required ICU care for septic shock, with mortality reaching 62% [6], highlighting the typically poor prognosis of community-acquired disease.

Optimal therapy for CRAB infections is challenging due to extensive resistance. A. baumannii strains resistant to carbapenems are usually non-susceptible to most standard antibiotics, often leaving only a few active options such as polymyxins (e.g., colistin) and, in rare instances, certain aminoglycosides or tetracyclines [7]. Current guidance emphasizes susceptibility-directed therapy and a combination of regimens for severe infections, and switching to oral alternatives when feasible [7]. In this case, the A. baumannii isolate was found to be susceptible to tobramycin and levofloxacin which guided the use of combination IV tobramycin and levofloxacin as empiric therapy, which was later switched to oral levofloxacin and doxycycline to complete the course once the patient improved. This is unlike other case reports where last-line agents such as colistin or high-dose sulbactam are used, often with poorer outcomes [7], [8]. Recent IDSA 2024 [9] guidance highlights cefiderocol and sulbactam–durlobactam as preferred options for CRAB infections when available. However, these agents were not accessible in our setting, limiting treatment options to available susceptibility-guided therapies

Although community-acquired pneumonia is typically treated with shorter antibiotic courses, a prolonged 6-week duration was selected in this case due to an atypical inflammatory trajectory. The patient initially demonstrated a favorable biochemical response, with CRP declining from > 200 mg/L at presentation to 12.5 mg/L by day 11. However, a subsequent rise in CRP to 142 mg/L on day 16, followed by persistently elevated levels (103 mg/L on day 23), raised concern for ongoing infection or incomplete eradication of a multidrug-resistant organism.

A key aspect of this case was the diagnostic approach. Bronchoalveolar lavage (BAL) proved to be invaluable in identifying the causative organism and its drug susceptibility profile. BAL sampling of the affected lung segment enabled definitive identification of A. baumannii and allowed timely antimicrobial susceptibility testing. In general, BAL is an effective diagnostic tool for pneumonia, as it allows direct sampling of lower respiratory secretions for organism isolation and susceptibility determination. Evidence suggests that BAL can improve pathogen detection and guide antibiotic selection, thereby helping determine the course of targeted therapy in pneumonia patients [10]. In our patient, the use of BAL led to the early identification of a carbapenem-resistant A. baumannii and guided the selection of tobramycin and levofloxacin, a tailored regimen that likely facilitated the favorable outcome.

## Conclusion

Overall, this case underscores several important points: CRAB remains a critical threat with high mortality in healthcare settings, and when it emerges as a cause of community-acquired pneumonia, prompt recognition and targeted treatment are vital given the infection’s rarity and potential severity. This case highlights the value of early invasive diagnostics and individualized, susceptibility-guided therapy, and contributes to the limited literature on community-acquired CRAB pneumonia.

## CRediT authorship contribution statement

**Hasaan salman shafi:** Writing – original draft, Methodology, Investigation, Conceptualization. **Omair Farooq:** Writing – review & editing, Project administration, Formal analysis.

## Consent

Written informed consent was obtained from the patient for the publication of this case report and accompanying clinical and radiological images.

## Ethical Statement

Written informed consent was obtained from the patient for publication of this case report and any accompanying images.

Ethical approval for this case report was obtained from the Institutional Ethics Review Committee (Approval dated 25 January 2026). All procedures performed were in accordance with the ethical standards of the institutional research committee and with the Declaration of Helsinki.

The patient’s identity has been kept confidential, and no identifying information has been disclosed.

## Funding Statement

This work received no specific grant from any funding agency in the public, commercial, or not-for-profit sectors.

## Declaration of Competing Interest

The authors declare no conflicts of interest related to this case report.
